# Leukoaraiosis Distribution and Cerebral Collaterals: A Systematic Review and Meta-Analysis

**DOI:** 10.3389/fneur.2022.869329

**Published:** 2022-06-24

**Authors:** Mangmang Xu, Wen Guo, Lucie Rascle, Laura Mechtouff, Norbert Nighoghossian, Omer Eker, Lu Wang, Nils Henninger, Abdul Ghani Mikati, Shihong Zhang, Bo Wu, Ming Liu

**Affiliations:** ^1^Center of Cerebrovascular Diseases, Department of Neurology, West China Hospital, Sichuan University, Chengdu, China; ^2^Department of Vascular Neurology, Hôpital Neurologique Pierre Wertheimer, Hospices Civils de Lyon, Lyon, France; ^3^Department of Neuroradiology of Pierre Wertheimer Hospital, Hospices Civils de Lyon, Lyon, France; ^4^Department of Rehabilitation Medicine, West China Hospital, Sichuan University, Chengdu, China; ^5^Department of Neurology and Department of Psychiatry, University of Massachusetts Chan Medical School, Worcester, MA, United States; ^6^Department of Neurosurgery, Tampa General Hospital, University of South Florida, Tampa, FL, United States

**Keywords:** leukoaraiosis, cerebral collaterals, Circle of Willis, meta-analysis, mechanical thrombectomy

## Abstract

**Background and Objective:**

Microvascular failure might result in the collapse of cerebral collaterals. However, controversy remains regarding the role of leukoaraiosis (LA) in collateral recruitment. We, therefore, performed a systematic review and meta-analysis of the association between LA and cerebral collaterals.

**Methods:**

Ovid Medline, PubMed, Embase, Web of Science, and three Chinese databases were searched from inception to August 2021. Two types of cerebral collaterals, including Circle of Willis (CoW) and leptomeningeal collaterals (LC), were investigated separately. Random effect models were used to calculate the pooled odds ratio (OR). Meta-regression and subgroup analyses were performed to explore the potential sources of heterogeneity.

**Results:**

From 14 studies (*n* = 2,451) that fulfilled our inclusion criteria, data from 13 could be pooled for analysis. Overall, there was a significant association between severe LA and incomplete CoW (pooled OR 1.66, 95% CI 1.18–2.32, *p* = 0.003), with low heterogeneity (*I*^2^ = 5.9%). This association remained significant in deep LA (pooled OR 1.48, 95% CI 1.04–2.11, *p* = 0.029, *I*^2^ = 0), but not periventricular LA. Similarly, there was a significant association between LA and LC (pooled OR 1.73, 95% CI 1.03–2.90, *p* = 0.037), but with high heterogeneity (*I*^2^ = 67.2%). Meta-regression indicated a negative association of sample size with the effect sizes (*p* = 0.029). In addition, most of the studies (7/9) included into the analysis of the relationship of severe LA with poor LC enrolled subjects with large vessel occlusion stroke, and this relationship remained significant when pooling the seven studies, but with high heterogeneity.

**Conclusion:**

Severe LA is associated with a higher prevalence of poor collaterals. This association is robust for CoW but weak for LC. Further studies are required to explore the underlying mechanisms.

## Introduction

The extent of cerebral collaterals is a key factor determining penumbral survival (1). Increasing evidence suggests that collateral circulation status predicts the progression of ischemic penumbra toward infarction, the final infarct volume (2), the risk for developing hemorrhagic transformation (3), and malignant infarction (4).

However, factors relating to the extent of cerebral collaterals remain only partially understood (5). One proposed mechanism is that microvascular failure during infarction results in the secondary collapse of macrovascular collaterals. It has been shown that ischemia and associated microvascular and glial injury cause microvascular lumen narrowing and an increase in blood viscosity and microvascular resistance (6). Moreover, the preclinical data indicated that leptomeningeal collateral (LC) recruitment is affected by vasodilatory responses of arterioles and microvasculature (7). Therefore, the pre-existing microvascular injury could conceivably exacerbate collateral collapse.

Small vessel disease related–leukoaraiosis (LA) is considered to represent ischemic damage due to chronic microvascular injury (8) and is associated with an increased risk of stroke (9) and poor functional outcomes after stroke (10, 11). Moreover, severe LA has been shown to increase the risk for hemorrhagic transformation after mechanical thrombectomy (MT) (12) and intravenous thrombolysis with recombinant tissue plasminogen activator (10, 13). Although both LA and collaterals are related to microvascular pathology, the association of LA with cerebral collaterals is only poorly understood and prior studies have yielded conflicting results (5, 14–16). Determining the role of LA in collateral recruitment may aid our understanding of the mechanisms that lead to collateral failure. To address this issue, we conducted a systematic review and meta-analysis to evaluate whether the severity and distribution of LA are associated with collateral status (based on direct and indirect qualitative grading scales) in patients with ischemic stroke and transient ischemic attack (TIA), or with atherosclerosis.

## Methods

This report was prepared with reference to the Preferred Reporting Items for Systematic Reviews and Meta-Analyses (PRISMA) (17).

### Search Strategy

We searched Ovid MEDLINE, PubMed, Embase, Web of Science, and three Chinese databases (China national knowledge infrastructure, WANFANG, and VIP) from inception to 9 August 2021 using a combination of search terms: “leukoaraiosis OR (white matter) OR (cerebral small vessel diseases)” AND “collateral,” without language restrictions. We also searched the reference lists for the relevant studies. Two authors (Mangmang Xu and Wen Guo) potentially identified relevant studies independently. The final list of included studies was decided on consensus. When additional study information was needed, we contacted the corresponding author of the original study whenever possible.

### Inclusion and Exclusion Criteria

Studies were included if they (1) investigated TIA or ischemic stroke, or atherosclerosis and (2) assessed cerebral collaterals and degree of LA. Exclusion criteria were as follows: (1) article that was not published in a peer-reviewed journal; (2) case reports; (3) not stroke or TIA or atherosclerosis; (4) conference abstract without available data; (5) reviews; and (6) animal studies.

### Data Extraction

Data were independently extracted by two authors (Mangmang Xu and Wen Guo) and cross-checked. For each included study, we extracted data on country; study sample size; demographic data including age, gender, hypertension, diabetes mellitus, and methods and techniques for determining LA and collaterals. We collected information on LA stratified by its distribution (periventricular, deep, and global white matter). The collateral type included Circle of Willis (CoW) and LC.

### Definitions

For the purpose of this study, severe periventricular LA was defined as Fazekas score 3 (5, 15, 18) or Fazekas scores 2 to 3 (19, 20) for periventricular regions. Severe deep LA was defined as Fazekas scores 2 to 3 (5, 15, 18–20) for deep regions. Global LA was defined when LA from both deep and periventricular white matter was combined into a single score in the original study. Specifically, severe global LA was defined as VSS≥3, (21, 22), or deep Fazekas scores 2 to 3, and/or periventricular Fazekas 3, (5), or total Fazekas score > 2, (20, 23), or as per the original study authors' definition (11, 15).

Poor LC was defined as follows: (1) Higashida score <3 (15, 18, 20, 22, 23), (2) less than contralateral hemisphere according to Lima et al. (24), (3) contrast filling <50% of the occluded territory according to Tan (5) for LC, or (4) no collateral filling as proposed by Angermaier et al. (25). Supplementary Table 1 summarizes the grading scores for LC collaterals and LA used in the included studies.

To determine the association between collateral status and LA, we included data on global LA. If no data on the global LA was available, we included deep LA when available, and periventricular LA when only periventricular LA was investigated (26). We also conducted separate investigations for the association of severe LA with incomplete CoW and poor LC, respectively. Finally, we conducted separate analyses for the association between collateral status with deep vs. periventricular LA.

Atherosclerosis was defined when a study enrolled patients with asymptomatic carotid atherosclerotic stenosis, carotid stenosis without information on incident stroke, or a clear diagnosis of clinically atherosclerosis disease in the original study.

### Quality Assessment

The risk of bias of included studies was assessed using the Newcastle–Ottawa Scale (Supplementary Table 2) (27). Age, hypertension, diabetes mellitus, and hyperlipidemia were defined as the important factors, which would define comparability for this scale (5, 28).

### Statistical Analysis

The risk of poor collateral status across studies was evaluated using random effects models and quantifying the association using odds ratios (ORs) with corresponding 95% CIs. We assessed heterogeneity using *I*^2^ statistics and the L'Abbé plot. The plot of summary outcome measures in the control group is on the *x*-axis, and the severe LA group is on the *y*-axis. Circles with larger sizes represent more precise and larger studies in the L'Abbé plot. Contour-enhanced funnel plot and Egger's test were used to detect the presence of publication bias (29). For the contour-enhanced funnel plot, 1%, 5%, and 10% significance were specified to produce the corresponding contour lines in the funnel plot. The study-level covariates which might affect the effect sizes were explored using subgroup meta-analysis (for categorical covariates) and meta-regression (for continuous covariates). Publication year, study sample size, mean age, male sex, hypertension, and diabetes mellitus were individually included in the meta-regression as covariates. The bubble plot was drawn to display the effect sizes against the continuous covariates with a *p*-value of < 0.05 in the meta-regression analyses.

In total, four subgroup analyses were performed to explore whether the following study-level categorical covariates could explain the observed heterogeneity: (1) country (USA vs. others); (2) MT treatment (yes vs. no); (3) LA assessment (Fazekas vs. others); and (4) modality for LA detection.

All the analyses were performed with Stata 16.0 (StataCorp LLC, College Station, TX).

## Results

### Study Characteristics

**Figure 1:**
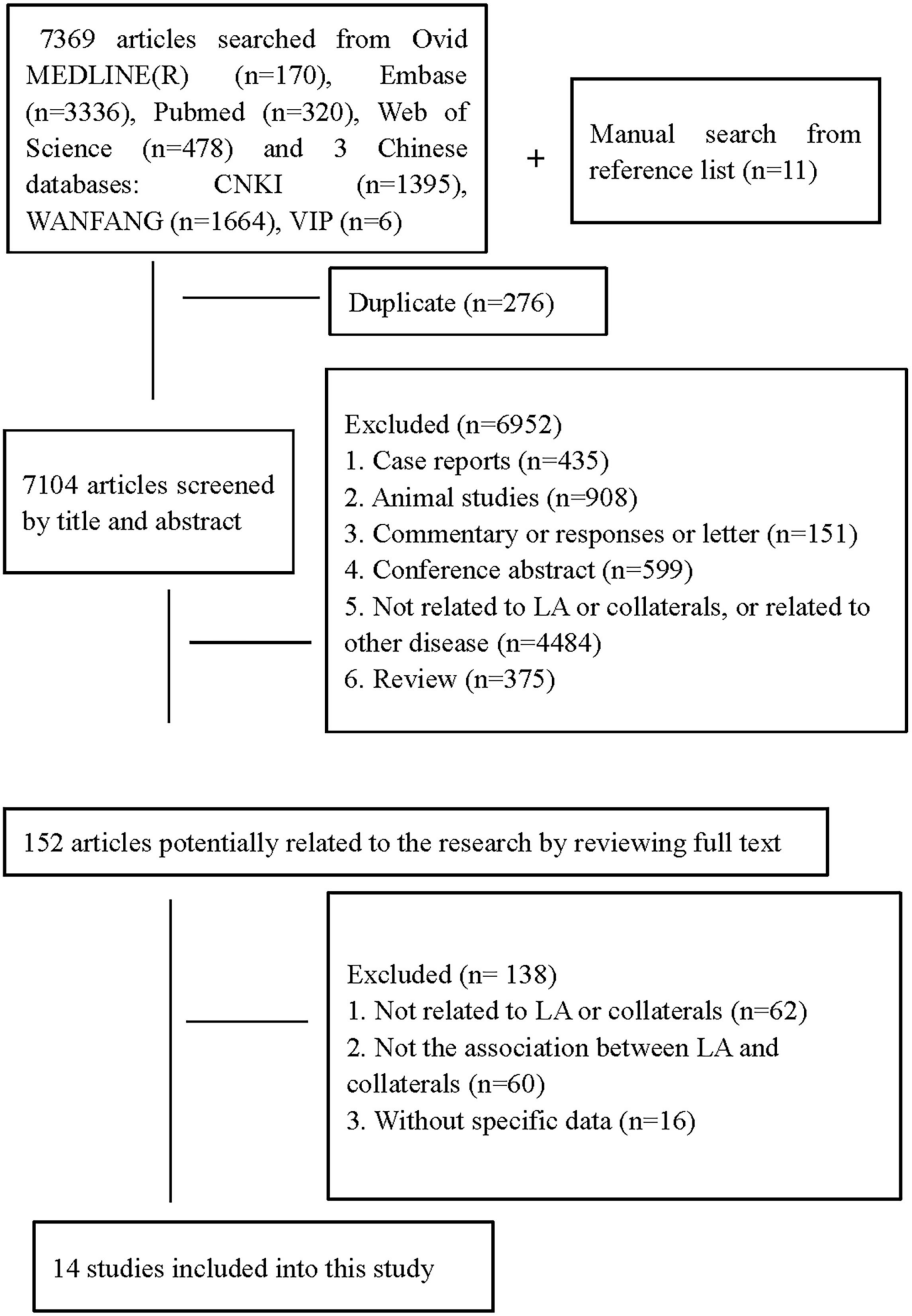
Flow chart of study selection.

We identified 7,369 citations in the search of databases and 11 citations by searching reference lists of relevant studies (Figure 1). In total, fourteen studies (5, 11, 14, 15, 18–23, 26, 30–32) with a total of 2,451 patients met our inclusion criteria. Ten studies assessed LA on MRI (5, 14, 15, 18, 19, 23, 26, 30–32), three on CT (20–22), and one on both CT and MRI (11). In total, four studied CoW (26, 30–32), nine assessed LC (5, 11, 14, 15, 18, 20–23), and one both LC and CoW (19). Details on demographic, vascular risk factors, and measured parameters of LA and collaterals across studies are summarized in Table 1. Of the included studies, Giurgiutiu et al. (14) used good collaterals as an outcome. The main results from this study were reviewed but excluded from the pooled analyses. Thus, data from 13 studies (5, 11, 15, 18–23, 26, 30–32) were pooled for the meta-analyses. Guo et al. (18) investigated collateral status separately for both hemispheres. For the present study, we only extracted data on the left hemisphere. Van der Grond (30) assessed CoW separately for the anterior and posterior parts, and we just included data on anterior CoW in line with most of our included studies investigating anterior circulation collaterals.

**Table 1:** Characteristics of the included studies.

**Study**	**Country**	**Sample, n** ** (%men)**	**Age, mean (SD)**	**HTN,** ***n* (%)**	**DM, n (%)**	**Population**	**LA**			**Collateral**			
							**Modality**	**Grading system**	**Definition of severe LA^**‡**^**	**Collateral type**	**Grading system**	**Modality**	**Definition of poor collaterals^**‡**^**
Duan (2014) (19)	China	333 (70.9)	70.9 (4.6)	260 (78.1)	85 (25.5)	TIA or AIS	MRI	Fazekas	dLA: deep Fazakas 2–3 pLA: periventricular Fazakas 2–3	CoW and LC	Not clear	DSA	Without opening of LC or CoW
Eker (2019) (15)	France	240 (50.8)	68.7 (16.1)	126 (52.5)	39 (16.2)	LVO AIS undergoing MT	MRI (1.5/3T)	Fazekas	dLA: deep Fazakas 2–3 pLA: periventricular Fazakas 3 gLA: Author's own definition	LC	Higashida Score	DSA	Higashida score < 3
Guo (2017) (18)	China	299 (68.6)	57 (13)	194 (64.9)	71 (23.7)	AIS with stenosis or occlusion in MCA M1 segment	MRI (3T)	Fazekas	dLA: deep Fazakas 2–3 pLA: periventricular Fazakas 3	LC	ASTIN/SIR (Higashida score)	DSA	Higashida score < 3;
Giurgiutiu (2015) (14)	USA	73 (43.8)	67.2 (15.7)	50 (68.5)	11 (22.9)	LVO AIS undergoing MT	MRI (1.5T)	LA volume	Continuous data	LC	Scale proposed by Souza et al.	CTA	Outcome was good collaterals
Henninger (2012) (21)	USA	87 (55)	67 (16)	62 (71)	13 (15)	AIS due to LVO	CT	VSS	gLA: VSS ≥ 3	LC	Scale proposed by Lima et al.	CTA	Absent or less than contralateral hemisphere;
Lin (2020) (5)	USA	100 (45)	64.6 (16.1)	73 (73.0)	23 (23.0)	LVO AIS undergoing MT	MRI (1.5/3T)	Fazekas	dLA: deep Fazakas 2–3 pLA: periventricular Fazakas 3 gLA: deep Fazakas 2–3/periventricular Fazakas 3	LC	The modified Tan score	CTA	Contrast filling < 50% of the occluded territory
Mark (2020) (20)	USA	178 (51.7)	67.7 (14.4)	128 (71.9)	48 (27.0)	LVO AIS undergoing MT	CT	Fazekas	Continuous data; Binary data: dLA: deep Fazakas 2–3 pLA: periventricular Fazakas 2–3 gLA: total Fazekas > 2	LC	ASTIN/SIR	CTA and DSA	Higashida score < 2
Mechtouf (2020) (23)	France	293 (54.6)	67.1 (16.2)	124/252 (49.2)	43/252 (17.1)	LVO AIS undergoing MT	MRI (1.5/3T)	Fazekas	gLA: total Fazekas > 2	LC	Higashida score	DSA	Higashida score < 3
Mikati (2020) (22)	USA	144 (51)	68 (57–81)	105 (73)	26 (18)	LVO AIS undergoing MT	CT	VSS	gLA: VSS ≥3	LC	Higashida score	DSA	Higashida score <3
Mutzenbach (2020) (11)	USA	209 (46.9)	75 (63–81)	132 (63.2)	29 (13.9)	LVO AIS undergoing MT	CT or MRI	ARWMC	gLA: the top 25 percentiles	LC	Scale proposed by Angermaier et al.	CTA	No collateral filling
van der Grond (2004) (30)	Netherlands	243 (86)	59 (10)	119 (49)	32 (13)	Clinically manifest atherosclerotic vessel disease	MRI (1.5T)	LA Lesion load	dLA: large confluent lesion	CoW	NA	MRA	Incomplete anterior part of CoW
Herweh (2012) (26)	Germany	14^†^ (57.1)	68.4 (6.5)	12 (85.7)	6 (42.9)	Asymptomatic internal carotid artery stenosis	MRI (3T)	Fazekas	pLA: periventricular Fazakas 2	CoW	NA	MRA	Incomplete CoW
Ye (2019) (31)	China	115 (81.7)	Not clear	82 (71.3)	52 (45.2)	Internal carotid artery stenosis	MRI (3T)	King et al.	dLA: OR, 95% CI pLA: OR, 95% CI	CoW	NA	MRA and Duplex ultrasound	Incomplete CoW
Lu (2017) (32)	China	123^§^ (54)	Not clear	(55.2)	(54.9)	Carotid artery stenosis	MRI (3T)	Fazekas	gLA: Deep and periventricular Fazakas 3	CoW	NA	DSA	Incomplete CoW

† *The original study included 15 patients into analysis, while 1 patient did not have data on collaterals*.

‡ *Per our prespecified definitions or the original author's own definition*.

§ *The original study included 315 patients with carotid atherosclerosis, and 123 patients have carotid stenosis and data of collaterals*.

*HTN, hypertension; DM, diabetes mellitus; NA, not applicable; LA, leukoaraiosis; SD, standard deviation; TIA, transient ischemic attack;dLA, deep LA; pLA, periventricular LA; gLA, global LA; CoW, Circle of Willis; LC, leptomeningeal collaterals; MT, mechanical thrombectomy; LVO, large vessel occlusion; MRI, magnetic resonance imaging; CT, computed tomography; MCA, middle cerebral artery; AIS, acute ischemic stroke; VSS, van Swieten scale; ARWMC, age-related white matter change*.

### Quality of Studies

All the included studies were cross-sectional in nature. For this reason, the non-response rate was not rated on the Newcastle–Ottawa scale. The quality of the studies was moderate (Newcastle–Ottawa scale scores ranging from 3 to 6 out of a maximum of 9) (Supplementary Table 2).

### Main Results

#### Association Between LA and CoW

**Figure 2:**
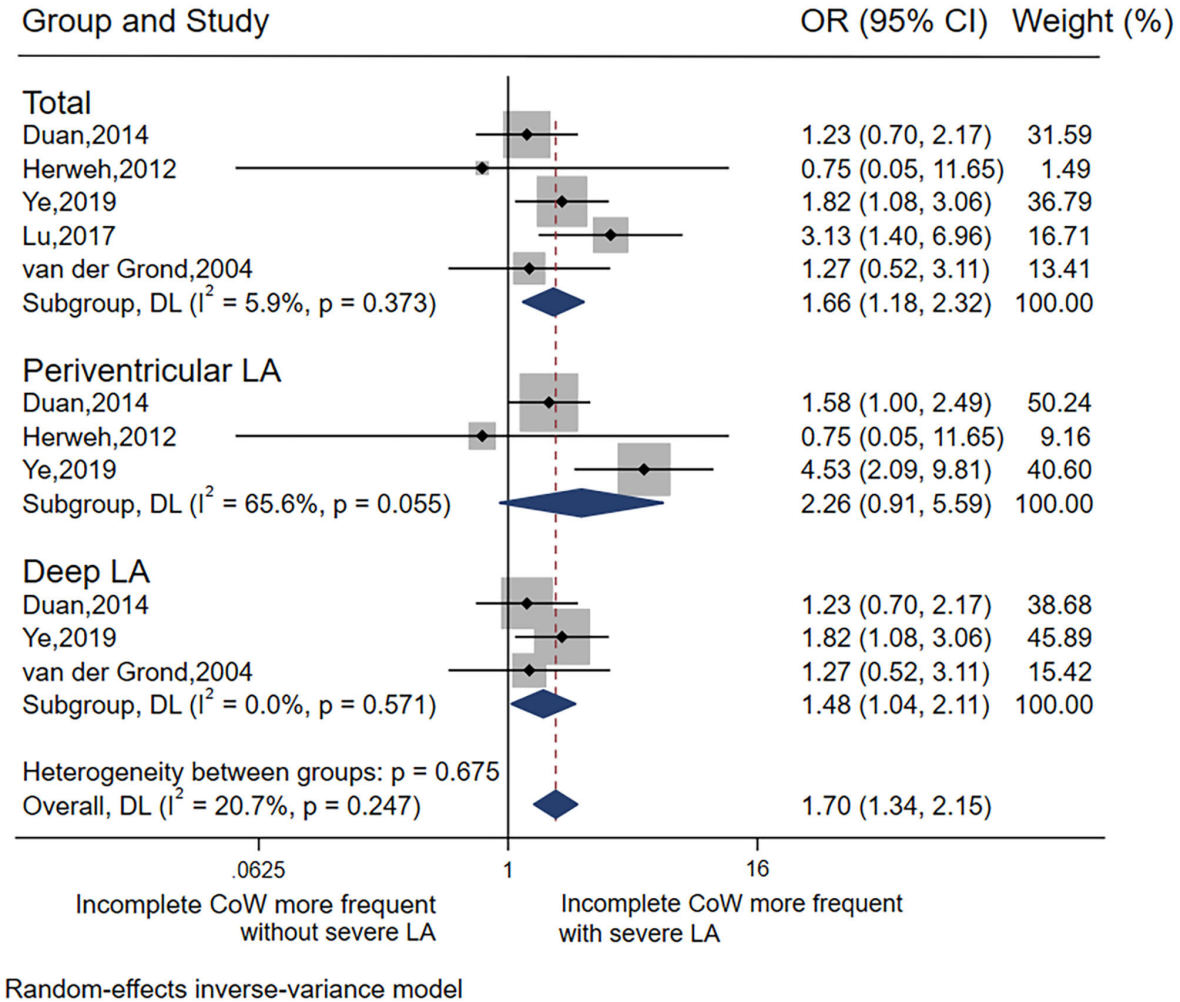
Forest plot for the association between LA distribution and CoW.

In total, five studies (19, 26, 30–32) were included in the analysis of LA and CoW. In general, there was a significant association between severe LA and incomplete CoW (pooled OR 1.66, 95% CI 1.18–2.32, *p* = 0.003), with low heterogeneity (*I*^2^ = 5.9%). This association remained significant for deep LA (pooled OR 1.48, 95% CI 1.04–2.11, *p* = 0.029) without heterogeneity (*I*^2^ = 0), but not periventricular LA (Table 2 Analysis 1; Figure 2). Of note, most of the studies (26, 30–32) included the analysis of the association between LA and CoW enrolled patients with atherosclerosis.

**Table 2:** Main results about the association between LA and collateral status.

	**Number of studies**	**Pooled estimate**		**Heterogeneity**
		**OR (95% CI)**	***P***	***I^**2**^***
**Analysis 1 LA and incomplete CoW**				
Total (19, 26, 30–32)	5	1.66 (1.18–2.32)	0.003	5.9%
Deep LA(19, 30, 31)	3	1.48 (1.04–2.11)	0.029	0
Periventricular LA(19, 26, 31)	3	2.26 (0.91–5.59)	0.077	65.6%
**Analysis 2 LA and poor LC**				
Total (5, 11, 15, 18–23)	9	1.73 (1.03–2.90)	0.037	67.2%
Deep LA (5, 15, 18–20)	5	1.81 (0.84–3.88)	0.129	77.3%
Periventricular LA (5, 15, 18–20)	5	1.78 (0.57–5.52)	0.319	86.7%
**Analysis 3 Multiple subgroup analyses for analysis 2: Total group (*****n*** **=** 9)^*^				
**Country (Test of group differences: Q**_**b**_**(1)** **=** **5.83, p** **=** **0.02)**				
Others (15, 18, 19)	3	0.91 (0.59–1.42)		0
USA (5, 11, 20–22)	5	2.66 (1.26–5.61)		66.5%
**MT treatment (Test of group differences: Q**_**b**_**(1)** **=** **0.45**, ***p*** **=** **0.50)**				
No (18, 19, 21)	3	1.36 (0.76–2.43)		0
Yes (5, 11, 15, 20, 22)	5	1.94 (0.82–4.63)		82.5%
**LA assessment (Test of group differences: Q**_**b**_**(1)** **=** **0.33**, ***p*** **=0.56)**				
Fazekas (5, 15, 18–20)	5	1.92 (0.80–4.62)		82.89%
Others (11, 21, 22)	3	1.41 (0.78–2.54)		0
**Modality for LA detection (Test of group differences: Q**_**b**_**(1)** **=** **0.46**, ***p*** **=0.50)**				
MRI(5, 15, 18, 19)	4	1.65 (0.56–4.81)		84.18%
CT (20–22)	3	2.54 (1.31–4.93)		26.38%

* *Data in study by Mechtouff et al. were extracted as OR and the corresponding 95% CI, rather than categorical variables, thus all the subgroup analyses did not include this study*.

*LA, leukoaraiosis; OR, odds ratios; CI, confidence intervals; CoW, Circle of Willis; LC, leptomeningeal collaterals; MT, mechanical thrombectomy; MRI, magnetic resonance imaging; CT, computed tomography*.

#### Association Between LA and LC

**Figure 3:**
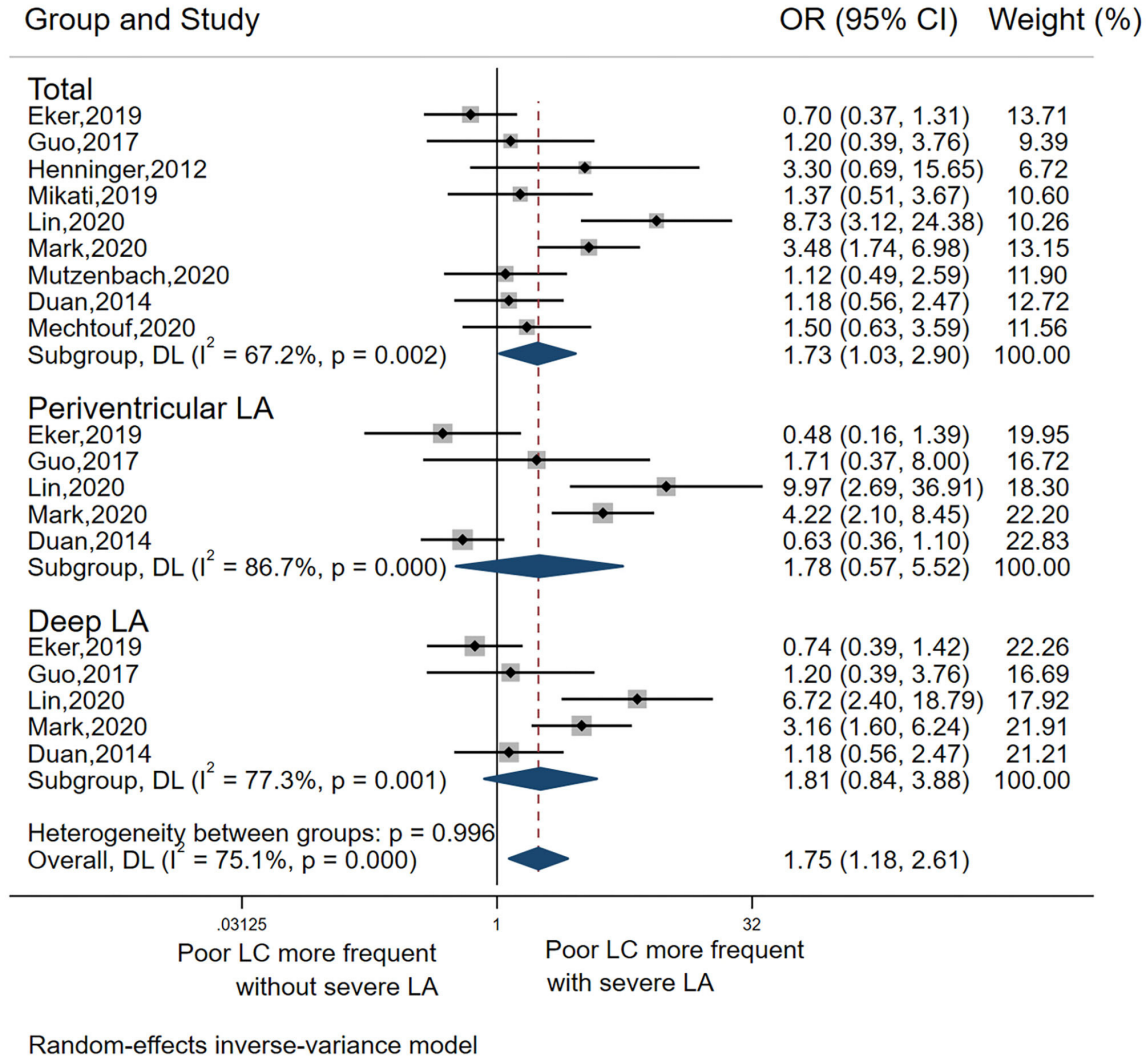
Forest plot for the association between LA distribution and poor LC.

**Figure 4:**
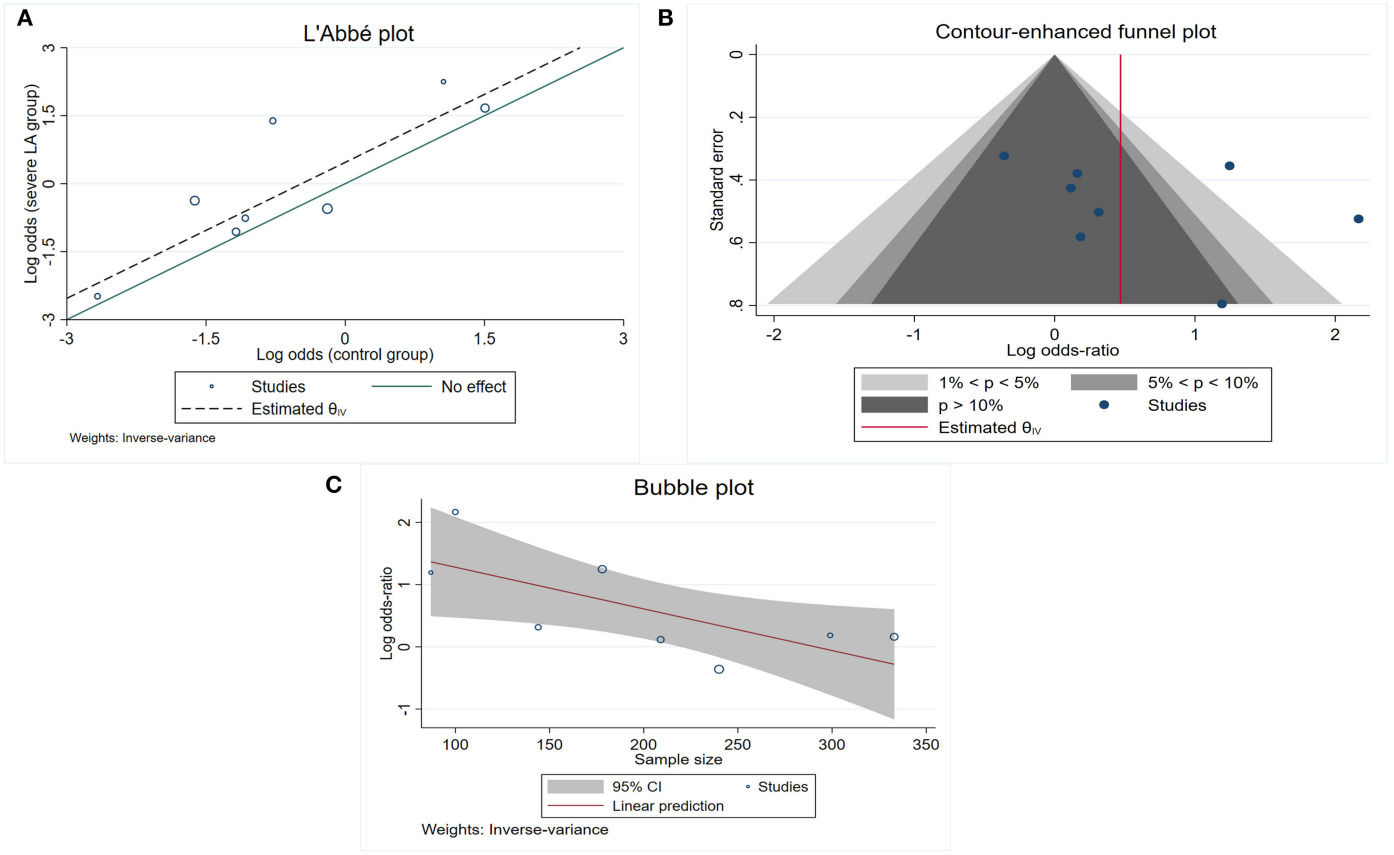
Study characteristics for those included into the association between total LA and poor LC. **(A)** L'Abb'e plot detects at least 1 study that is far away from the effect-size line (the dashed line); **(B)** contour-enhanced funnel plot shows that the majority of included studies are nonsignificant, indicating that other factors, other than publication bias, need to be considered for the asymmetry; **(C)** bubble plot shows that the effect sizes decreased as the number of sample size increased.

The pooled OR from the nine included studies (5, 11, 15, 18–23) indicated a significant association between LA and LC (pooled OR 1.73, 95% CI 1.03–2.90, *p* = 0.037) but with high heterogeneity *I*^2^ = 67.2%) (Table 2, Analysis 2; Figure 3). Also, the L'Abbé plot (Figure 4A) detects at least 1 study that is far away from the effect-size line (the dashed line), supporting the presence of heterogeneity in these data. Interestingly, the majority of studies (7 out of 9) that were included in the analysis of LA and LC enrolled patients with large vessel occlusion (LVO) stroke. The association remained significant (pooled OR 1.95, 95% CI 1.01–3.77, *p* = 0.046) based on pooled data from the seven studies (5, 11, 15, 20–23), but also with high heterogeneity (*I*^2^ = 74.3%). The study by Giurgiutiu (14), which we could not include in the pooled analysis, found that the LA volume was inversely associated with a good collateral grade of LC in patients with LVO stroke even after correcting for confounding factors (i.e., the prevalence of poor LC increased with higher LA volumes).

Contour-enhanced funnel plot (Figure 4B) and Egger's test (*z* = 0.99, *p* = 0.321) did not indicate a significant publication bias. In the meta-regression, the sample size was a potential source of heterogeneity (*p* = 0.029). Bubble plot analysis indicated that the effect sizes decreased with increasing sample size (Figure 4C). When entered separately into meta-regression, publication year, mean age, male sex, hypertension, and diabetes mellitus were not significantly associated with effect size. Multiple subgroup analyses (Table 2, Analysis 3) found that country was a potential source of heterogeneity (*p* = 0.02). Although subgroup analyses of other categorical variables such as MT, LA assessment, and modality for LA detection did not show any significant difference between groups, these considered grouping might help explain some of the observed between-study heterogeneity. We found that MT treatment (vs. no MT treatment), LA assessment by Fazekas (vs. LA assessment by another assessment method), and LA detection using MRI (vs. CT) were associated with greater heterogeneity.

Among the nine studies that assessed the association between LA and LC, five (5, 15, 18–20) investigated both deep LA and periventricular LA. In these five studies, there was no significant association between poor collaterals with either severe deep LA or periventricular LA (Table 2 Analysis 2; Figure 3).

## Discussion

This meta-analysis of 13 studies, together with the review of one study which could not be included in the pooled analyses, indicated that severe LA was associated with an increased risk for either incomplete CoW or poor LC, especially for incomplete CoW. When stratified by LA distribution, incomplete CoW was associated with deep LA, but not periventricular LA.

Nevertheless, we found substantial heterogeneity in the association between LA and LC. This heterogeneity was related to sample size and study country according to the results of meta-regression and subgroup analyses. Also, both the pooled lower 95% CI for the association between LA and LC (1.03) and the subgroup analysis of this association in LVO (1.01) was close to 1, indicating that the relationship was weak statistically. The different relationship strengths of LA with CoW vs. LC might be explained by the different study populations in CoW analysis and LC analysis. Most of the studies (4/5) included in the analysis of incomplete CoW enrolled patients with atherosclerosis. While the majority of studies (7/9) entered in the analysis of poor LC enrolled LVO stroke, and among the included LVO studies that reported on the stroke mechanism (more frequently cardioembolic [44~55%] than large artery atherosclerosis related [11~23%]) (11, 21, 22), there was a negative association between LA and poor LC. This suggests that the stroke mechanism may play a role in the association of LA and collaterals with an overall weaker association between the impact of LA on collaterals in stroke due to cardioembolism than in large artery atherosclerosis. Arguably, this difference is related to the slowly progressive nature of intracranial arterial stenosis, which could allow for the development of more effective compensatory collaterals, as compared to artery occlusion due to cardioembolism (33, 34), and is also related to the finding that hypoperfusion alone caused by the large artery occlusion does not seem to contribute to LA (35). However, our study design does not allow us to draw firm conclusions in this regard as none of the included studies stratified the association of collaterals with LA by the stroke subtype. Thus, further studies are required to clarify this issue.

We noticed that an incomplete CoW was associated with severe deep LA, but not periventricular LA when stratified by LA distribution. However, due to the small number of studies, it was not possible to perform a sensitivity analysis or meta-regression to detect the source of heterogeneity. Future studies with larger sample sizes are warranted to investigate the role of periventricular LA in CoW.

The findings of our study are of potential clinical significance. Mitigating the progression of LA may benefit patients at high risk for stroke, such as in the setting of large artery atherosclerosis, by maintaining collateral recruitment. It has been hypothesized that the collapse of collateral flow might be secondary to microvascular resistance during the ischemic process. Indeed, pre-existing small vessel wall alterations might result in increased vascular resistance within penetrating arterioles, therefore, hampering collateral flow reversal (6). Furthermore, the development of leptomeningeal vessels and particularly the opening of cerebral collaterals likely depends upon several compensatory hemodynamic, metabolic, and neural mechanisms (28), and also the inflammatory processes (36). Hence, it is important to define the role of microcirculation damage in collateral flow recruitment and the underlying factors, such as inflammatory process, vascular risk factors, and neural mechanisms to modify this association in future studies. Thus, targeting these factors may be a promising strategy to improve collateral status in the setting of an acute stroke. A better understanding of these factors, how they relate to LA and collateral status is critical as there is presently no specific drug for the treatment of LA. Current therapy rests on optimizing modifiable risk factors, in particular by controlling hypertension and statin use, which may be particularly beneficial in the setting of severe LA (37).

Strengths of our study include a comprehensive search strategy, use of widely accepted grading systems to assess LA and collaterals in the included studies, and our approach to conducting meta-regression by separately including publication year, sample size, mean age, male sex, hypertension, and diabetes mellitus as covariates and subgroup analyses stratified by country, MT treatment, and LA assessment, respectively. Limitations of our study relate to the fact that the pooled sample size for analyses stratified by LA distribution and subgroup analyses was only modest, reducing the power of interpretation. Thus, our results require confirmation by future studies. Due to the nature of visual LA rating scales, automated methods to assess LA volume may increase reliability. With regard to the imaging modality for LA detection, MRI is superior to CT. However, subgroup analysis did not find a significant difference in the association of LC with LA between subgroups that used MRI and those that used CT to assess LA in our present study, in accordance with the previous evidence that CT and MRI had equal detectability of severe LA (8). It is possible that we did not identify all the relevant studies, which may have introduced selection bias. However, our comprehensive search approach included multiple online databases, conference abstracts, and also hand-searching for relevant references for additional studies, and emailing authors for additional data assuage concerns in this regard. Our results should be interpreted with caution. It could not be generalized to all the patients with ischemic stroke or normal population, since most of the included studies enrolled patients with atherosclerosis or LVO stroke.

## Conclusion

In summary, severe LA was associated with a greater prevalence of poor collaterals. This association was robust for LA with CoW, but weak for LA with LC. Further studies are required to explore the underlying mechanisms.

## Data Availability Statement

Data will be made available on reasonable request. Access requests should be directed to the corresponding author(s).

## Author Contributions

MX and ML designed the study. MX and WG conducted the literature search, extracted data, and draft this manuscript. MX and LW analyzed the data. MX, NN, NH, and ML interpreted the data. LR, LM, LW, AM, and OE reviewed and made substantial improvements for this work. NN and NH review, edit, and gave important suggestions. SZ, BW, and ML made important suggestions and revised manuscript. All authors contributed to the article and approved the submitted version.

## Funding

This study was supported by National Natural Science Foundation of China (81974181, 82001250, and 81974208), China Postdoctoral Science Foundation (2020M683322, 2021T140488), and the 1.3.5 Project for Disciplines of Excellence, West China Hospital, Sichuan University (ZYGD18009). Dr. NH was supported by K08NS091499 from the National Institute of Neurological Disorders and Stroke of the National Institutes of Health during the conduct of the study. The content is solely the responsibility of the authors and does not necessarily represent the official views of the National Institutes of Health.

## Conflict of Interest

The authors declare that the research was conducted in the absence of any commercial or financial relationships that could be construed as a potential conflict of interest.

## Publisher's Note

All claims expressed in this article are solely those of the authors and do not necessarily represent those of their affiliated organizations, or those of the publisher, the editors and the reviewers. Any product that may be evaluated in this article, or claim that may be made by its manufacturer, is not guaranteed or endorsed by the publisher.
