# Supplementary material for: Leukoaraiosis Distribution and Cerebral Collaterals: A Systematic Review and Meta-Analysis
Source: Front Neurol. 2022 Jun 24;13:869329. doi: 10.3389/fneur.2022.869329 (PMC9263359; doi:10.3389/fneur.2022.869329)
Supplement: Supplementary file 1 [file Table_1.docx]

**Supplementary table 1 Grading scores for collaterals and leukoaraiosis**

| **Scale name** | **Grading system** |
| --- | --- |
| **Collaterals** | |
| Higashida score[^1^](#_ENREF_1) | 0: No collateral vessels filled  1: Slow collateral filling to periphery  2: Rapid collateral filling to periphery  3: Collaterals with slow but complete flow in ischaemic bed  4: Rapid and complete flow in entire ischaemic territory |
| Collateral score system proposed by Souza et al[^2^](#_ENREF_2) | 0: Absent collaterals >50% of an M2 territory  1: Diminished collaterals >50% M2 territory;  2: Diminished collaterals <50% M2 territory;  3: Collaterals equal to contralateral side;  4: Increased collaterals. |
| Scale proposed Lima et al[^3^](#_ENREF_3) | 1: Absent  2: Less than contralateral hemisphere  3: Equal to contralateral hemisphere  4: Greater than contralateral hemisphere  5: Exuberant |
| Angermaier et al[^4^](#_ENREF_4) | 0: no collateral filling  1: =<50%  2: =>50% but <100%  3: =100% collateral filling |
| Modified Tan et al[^5^](#_ENREF_5) | 0: Absent  1: collaterals filling ≤50% of the occluded territory  2:>51–99% of the occluded territory  3: 100% of the occluded territory |
| **Leukoaraiosis** | |
| Fazekas et al[^6^](#_ENREF_6) | **Periventricular hyperintensity**  0=absence, 1=“caps” or “pencil-thin” lining, 2=smooth “halo”, 3= irregular PVH extending into the deep white matter  **Deep white matter hyperintensity**  0=absence, 1= punctate foci, 2=beginning confluence of foci, 3=large confluent areas |
| VSS[^7^](#_ENREF_7) | 0: No lesions  1: The abnormality was restricted to the region adjoining the ventricles  2: The increased hypodensity involved the entire region from lateral ventricles to the cortex |
| ARWMC[^8^](#_ENREF_8) | **White matter lesions**  0: No lesions (including symmetrical, well-defined caps or bands)  1: Focal lesions  2: Beginning confluence of lesions  3: Diffuse involvement of the entire region, with or without involvement of U fibers  **Basal ganglia lesions**  0: No lesions  1: 1 focal lesion (≥5 mm)  2: >1 focal lesion  3: Confluent lesions |
| King et al.[^9^](#_ENREF_9) | **Periventricular hyperintensity**  The distance perpendicular from the ventricle in the axial plane:  0:< 3 mm,  1:3 to 10 mm,  2:10 to 20 mm,  3:≥ 20 mm  **Deep hyperintensity**  The largest diameter:  0:< 3 mm for all lesions  1: 3 to 10 mm for a single lesion or < 20 mm for grouped lesions,  2: 10 to 20 mm for a single lesion or > 20 mm for grouped lesions,  3: >20 mm for a single or confluent lesion. |

**References:**

1. Higashida RT, Furlan AJ, Roberts H, et al. Trial design and reporting standards for intra-arterial cerebral thrombolysis for acute ischemic stroke. *Stroke*. 2003; 34: e109-37.

2. Souza LC, Yoo AJ, Chaudhry ZA, et al. Malignant CTA collateral profile is highly specific for large admission DWI infarct core and poor outcome in acute stroke. *AJNR American journal of neuroradiology*. 2012; 33: 1331-6.

3. Lima FO, Furie KL, Silva GS, et al. The pattern of leptomeningeal collaterals on CT angiography is a strong predictor of long-term functional outcome in stroke patients with large vessel intracranial occlusion. *Stroke*. 2010; 41: 2316-22.

4. Angermaier A, Langner S, Kirsch M, Kessler C, Hosten N and Khaw AV. CT-angiographic collateralization predicts final infarct volume after intra-arterial thrombolysis for acute anterior circulation ischemic stroke. *Cerebrovascular diseases (Basel, Switzerland)*. 2011; 31: 177-84.

5. Tan JC, Dillon WP, Liu S, Adler F, Smith WS and Wintermark M. Systematic comparison of perfusion-CT and CT-angiography in acute stroke patients. *Annals of neurology*. 2007; 61: 533-43.

6. Fazekas F, Chawluk JB, Alavi A, Hurtig HI and Zimmerman RA. MR signal abnormalities at 1.5 T in Alzheimer's dementia and normal aging. *AJR American journal of roentgenology*. 1987; 149: 351-6.

7. van Swieten JC, Hijdra A, Koudstaal PJ and van Gijn J. Grading white matter lesions on CT and MRI: a simple scale. *Journal of neurology, neurosurgery, and psychiatry*. 1990; 53: 1080-3.

8. Wahlund LO, Barkhof F, Fazekas F, et al. A new rating scale for age-related white matter changes applicable to MRI and CT. *Stroke*. 2001; 32: 1318-22.

9. Ye H, Wu X, Yan J, Wang J, Qiu J and Wang Y. Completeness of circle of Willis and white matter hyperintensities in patients with severe internal carotid artery stenosis. *Neurological sciences : official journal of the Italian Neurological Society and of the Italian Society of Clinical Neurophysiology*. 2019; 40: 509-14.
