# Supplementary material for: Leukoaraiosis Distribution and Cerebral Collaterals: A Systematic Review and Meta-Analysis
Source: Front Neurol. 2022 Jun 24;13:869329. doi: 10.3389/fneur.2022.869329 (PMC9263359; doi:10.3389/fneur.2022.869329)
Supplement: Supplementary file 2 [file Table_2.DOCX]

**Supplementary table 2 Assessment of quality of included studies using Newcastle-Ottawa scale (case control studies)**

| **Article** | **Selection** | | | | **Comparability** | **Exposure** | | | **Score** |
| --- | --- | --- | --- | --- | --- | --- | --- | --- | --- |
|  | 1) | 2) | 3) | 4) | 1) | 1) | 2) | 3) |  |
| Duan (2014)^19^ | * | * |  |  |  | * | * |  | 4 |
| Eker (2019)^15^ | * | * |  |  | ** | * | * |  | 6 |
| Guo (2017)^18^ | * | * |  |  |  | * | * |  | 4 |
| Giurgiutiu (2015)^14^ | * | * |  |  | * | * | * |  | 5 |
| Henninger (2012)^21^ | * | * |  |  |  | * | * |  | 4 |
| Lin (2020)^5^ | * | * |  |  | ** | * | * |  | 6 |
| Mark (2020)^20^ | * | * |  |  | * | * | * |  | 5 |
| Mechtouf (2020)^23^ | * | * |  |  |  | * | * |  | 4 |
| Mikati (2020)^22^ | * | * |  |  |  | * | * |  | 4 |
| Mutzenbach (2020)^11^ | * | * |  |  |  | * | * |  | 4 |
| van der Grond (2004)^30^ | * | * |  |  |  | * | * |  | 4 |
| Herweh (2012)^26^ | * | * |  |  |  | * | * |  | 4 |
| Ye (2019)^31^ | * | * |  |  | ** | * | * |  | 6 |
| Lu (2017)^32^ | * |  |  |  |  | * | * |  | 3 |

Selection: 1) Is the case definition adequate? 2) Representativeness of the cases; 3) Selection of Controls; 4) Definition of Controls

Comparability: 1) Comparability of cases and controls on the basis of the design or analysis

Exposure: 1) Ascertainment of exposure; 2) Same method of ascertainment for cases and controls; 3) Non-Response rate
